# Supplementary material for: Predicting visual function by interpreting a neuronal wiring diagram
Source: Nature. 2024 Oct 2;634(8032):113–23. doi: 10.1038/s41586-024-07953-5 (PMC11446822; doi:10.1038/s41586-024-07953-5)
Supplement: Supplementary file 6 — CRF and ERF predictions for individual TmY4 and TmY9 cells. Analogous to Supplementary Data 3, but for TmY target types. Shown are the top four monosynaptic pathways, the strongest pathway passing through each of the top ten intermediary types (ranking from Extended Data Fig. 7), and the trisynaptic pathway Tm1–TmY–Dm3–TmY (see the section entitled Prediction of spatial normalization). [file 41586_2024_7953_MOESM6_ESM.zip › DataS4/TmY9qΓèÑ/720575940617250233.pdf]

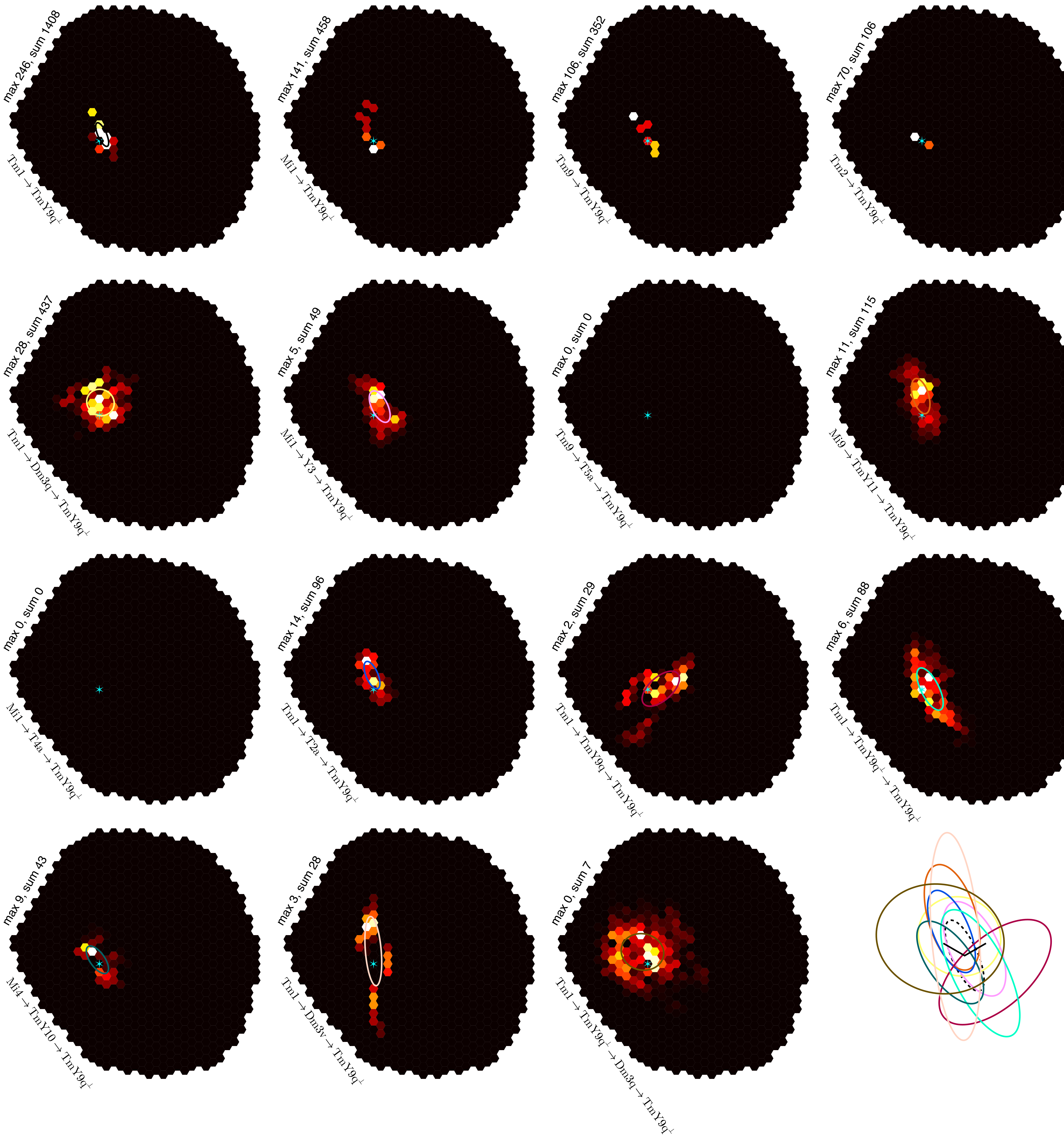

max 246, sum 1408
Tm1 → TmY9q⊥
max 141, sum 458
Mi1 → TmY9q⊥
max 106, sum 352
Tm9 → TmY9q⊥
max 70, sum 106
Tm2 → TmY9q⊥
max 28, sum 437
Tm1 → Dm3q → TmY9q⊥
max 5, sum 49
Mi1 → Y3 → TmY9q⊥
max 0, sum 0
Tm9 → T5a → TmY9q⊥
max 11, sum 115
Mi9 → TmY11 → TmY9q⊥
max 0, sum 0
Mi1 → T4a → TmY9q⊥
max 14, sum 96
Tm1 → T2a → TmY9q⊥
max 2, sum 29
Tm1 → TmY9q → TmY9q⊥
max 6, sum 88
Tm1 → TmY9q⊥ → TmY9q⊥
max 9, sum 43
Mi4 → TmY10 → TmY9q⊥
max 3, sum 28
Tm1 → Dm3v → TmY9q⊥
max 0, sum 7
Tm1 → TmY9q⊥ → Dm3q → TmY9q⊥
